# Cryogenic Electron Microscopy Methodologies as Analytical Tools for the Study of Self-Assembled Pharmaceutics

**DOI:** 10.3390/pharmaceutics13071015

**Published:** 2021-07-02

**Authors:** Na’ama Koifman, Yeshayahu Talmon

**Affiliations:** Department of Chemical Engineering and The Russell Berrie Nanotechnology Institute, Technion-Israel Institute of Technology, Haifa 3200003, Israel; naamae@technion.ac.il

**Keywords:** drug delivery, cryo-TEM, cryo-SEM, vesicles, liposomes, self-assembly

## Abstract

Many pharmaceutics are aqueous dispersions of small or large molecules, often self-assembled in complexes from a few to hundreds of molecules. In many cases, the dispersing liquid is non-aqueous. Many pharmaceutical preparations are very viscous. The efficacy of those dispersions is in many cases a function of the nanostructure of those complexes or aggregates. To study the nanostructure of those systems, one needs electron microscopy, the only way to obtain nanostructural information by recording direct images whose interpretation is not model-dependent. However, these methodologies are complicated by the need to make liquid systems compatible with high vacuum in electron microscopes. There are also issues related to the interaction of the electron beam with the specimen such as micrograph contrast, electron beam radiation damage, and artifacts associated with specimen preparation. In this article, which is focused on the state of the art of imaging self-assembled complexes, we briefly describe cryogenic temperature transmission electron microscopy (cryo-TEM) and cryogenic temperature scanning electron microcopy (cryo-SEM). We present the principles of these methodologies, give examples of their applications as analytical tools for pharmaceutics, and list their limitations and ways to avoid pitfalls in their application.

## 1. Introduction

Cryogenic temperature electron microscopy (cryo-EM) is a set of sophisticated tools for the imaging of nanostructured liquids, and, as such, is most useful for the nanostructural characterization of a wide range of drug delivery systems. Depending on the characteristics of the drug delivery system, one can choose which direct imaging method to use. Size, viscosity, and the required resolution are some of the parameters that affect the method of choice. Hernandez et al. [1] used cryogenic temperature transmission electron microscopy (cryo-TEM) as the main tool for the study of nanobubbles, namely, of lipid/polymer-stabilized gas-containing nanostructures, which can be triggered externally by ultrasound, which makes them attractive candidates for drug delivery applications. The use of cryo-TEM helped determine whether gas remained intact in nanobubbles or was released upon ultrasound flash. Erlich et al. [2] used cryogenic temperature scanning electron microscopy (cryo-SEM) to characterize microencapsulated hydrophobic drugs by the sol–gel process. They reported excellent preservation of the structures using the cryo-SEM specimen preparation procedure, while the combination of different HR-SEM detectors allowed full understanding of the system nanostructure.

Extracellular vesicles (EVs), namely, vesicles or liposomes released from the cell cytoplasm or from the cell membrane, have been studied mostly for their basic scientific biological significance, for their importance in disease mechanism and detection, and for their potential use as drug delivery vectors [3,4,5,6]. Direct imaging of EVs using cryo-EM is an important tool in answering some substantial questions regarding EVs: what the subclasses of EVs are, how isolation methods affect isolated EVs, and how EVs of different origins vary, to name a few [7,8]. Matthies et al. used cryo-TEM and cryo-tomography to study EVs isolated from primary cortical neurons. The 3D characterization by cryo-tomography revealed the existence of macromolecular clusters at specific positions inside these EVs, which may play a role in signaling [9]. Wolfram et al. used cryo-TEM to assess the nanostructure of EVs of different origins and their biological functions [10,11].

Another type of biomimetic nanovesicles is termed “nano-ghosts.” These vesicles are prepared from membranes of mesenchymal stem cells, can be labeled and loaded with drugs, and serve as drug delivery carriers [12]. Their characterization must include cryo-TEM since samples pf nano-ghosts may include protein aggregates and other cellular matter, which could only be differentiated from empty nano-ghosts by direct imaging.

In recent years, cryo-EM has become an important tool in structural biology, especially in structure determination of small molecules, mostly of proteins, by what is known as single particle analysis (SPA). The progress in cryo-EM, mostly in implementing direct detector cameras, but also in automation of specimen transfer and image acquisition, allowed high-resolution determination of these molecules and set cryo-EM as a powerful tool in drug discovery [13,14,15,16]. As long as cryo-EM specimen preparation preserves molecules in their native state, it enables high-resolution structural research of a variety of proteins in a straightforward manner. Additionally, it allows the imaging of large complexes, e.g., several proteins or proteins and drugs, and not only of pure isolated proteins, which is very valuable in structure-based drug discovery. Cryo-EM often detects conformational changes, which can help determine new binding sites for potential drugs or contribute to the understanding of the biological mechanism or the function the protein serves. Recently, following the COVID-19 outbreak, intensive cryo-EM work allowed structure determination of the virus and contributed significantly to the understanding of the structure of its spike proteins, their orientations and potential functions [17]. Ke et al. used cryo-electron tomography (cryo-ET), a methodology that reconstructs the three-dimensional structure of an object from a tilt-series of two-dimensional projections, to image SARS-CoV-2 viruses. They combined it with single particle analysis electron tomography for high-resolution structure determination of intact spike proteins. This work was very valuable for COVID-19 researchers and, because these proteins can, in certain conformations, protect the virus from the body’s immune response, this information was crucial for vaccine development [18]. Another advanced tool that has been used both for SPA and cryo-TEM imaging is the “Volta phase plate” which enhances the image contrast by converting phase differences into amplitude differences while working at the exact focus or at a slight underfocus of the TEM objective lens. It is very useful for imaging low-contrast systems at high resolution. Liang et al. [19] used phase plate cryo-EM for structure determination of a class B G-protein-coupled receptor (GPCR), a therapeutic target for a range of bone diseases. The use of Volta phase plate cryo-EM in imaging activated complexes as little as 150 kDa, leading to near-atomic resolution.

Many pharmaceutical preparations are based on hydrogels that are also potential platforms for tissue engineering, serving as scaffolds for cell growth. Cryo-SEM has been used to characterize hydrogel pore size and nanostructure. However, these data are often marred by dehydration and freezing artifacts because of the specimen preparation methods used [20,21,22]. The possible artifacts that result from different specimen preparation methods (plunging into liquid nitrogen slush or liquid ethane, high-pressure freezing) for cryo-SEM imaging of hydrogels, as well as from the recommended approaches, are described by Aston et al. [23]. Marmorat et al. examined the nanostructure of a crosslinked gelatin-based hydrogel by cryo-SEM, demonstrating differences in mesh sizes of samples with different crosslinking levels. The secondary structure of gelatin fibers was also preserved and imaged due to the hydrated state of the specimen [24].

Liposomes are widely studied and used as drug delivery systems, given their ease of preparation, biocompatibility derived from their phospholipid composition mimicking natural cell membranes, and versatility in their ability to encapsulate a variety of molecules, either in their core or in their membrane [25,26]. Cryo-TEM is an effective means for the characterization of liposomal systems because the membrane and the core of a liposome are well-resolved [27,28]. One outstanding example is the Doxil^®^ system, which was the first nanodrug to be approved by the FDA in 1995, encapsulating the anticancer drug, doxorubicin, in PEGylated liposomes, turning them into “stealth” liposomes with prolonged circulation times [29]. Another potential approach to personalized cancer treatment is loading liposomes with anticancer drugs and short DNA “barcodes” for specific detection of the drugs that are most effective in eradicating tumor cells [30].

While liposome systems are not thermodynamically stable, microemulsions consist of water, oil, and surfactants and self-assemble into nanometer-sized droplets or a bicontinuous thermodynamically stable phase. Microemulsions have several advantages as potential drug carriers, including their transparency, long term stability, and ease of preparation [31,32]. For drug delivery purposes, microemulsions are mostly used for solubilizing hydrophobic drugs (oil-in-water microemulsions, O/W), improving their delivery in aqueous media and thus decreasing the total administered dose. Water-in-oil (W/O) microemulsions are well-suited for delivery of water-soluble drugs, and the existence of both the hydrophilic phase and the hydrophobic phase allows incorporating both types of drugs [33]. The small domain size in microemulsions and ease of solubilization and absorption in the digestive system make them suitable for oral delivery, while their transparent quality contributes to their use in ocular administration. Direct imaging of microemulsions can be performed by either cryo-TEM [34,35], cryo-SEM [36], or both, depending on the viscosity of the formulation. Nanostructural characterization of microemulsions along a specific dilution line or at different compositions of oil and water may require complementarity of both cryo-EM methods, as demonstrated in the study of Davidovich et al. [37]. More information on cryo-EM of microemulsions and the connection between the systems’ nanostructures and properties is given in a recent review by Gradzielski et al. [38].

Another type of complex aggregates with high potential in drug delivery in whole and in medical theranostic application in particular are cubosomes [39,40]. For those systems, cryo-TEM has been proven most useful in the characterization of the preparation, showing the coexistence of simple vesicles with liposomes in the system, as well as the existence of an intermediate state of aggregation known as “interlamellar attachments,” which had been shown earlier by cryo-TEM [41].

Cryo-TEM has also been used to follow the effect of various factors on drug preparation in vitro and in vivo. Quite often, a drug is prepared for IV administration by dilution in a physiological solution. This may cause drastic changes in the state of aggregation of the active component in the liquid phase, which may have adverse effects on the patient. An example was given by Szebeni et al. [42], who showed with direct imaging using cryo-TEM the formation of complement-activating particles in aqueous solutions of the anticancer drug paclitaxel (Taxol) and their possible role in hypersensitivity reactions to this drug. Attili-Qadri et al. [43] took cryo-TEM a step further by using it to monitor paclitaxel in blood circulation of rats following oral delivery. The reader may also find the review by Kuntsche et al. [44] interesting.

Although cryo-TEM single particle analysis used for drug discovery is an important topic, it is outside the scope of this publication.

## 2. Cryogenic-Temperature Electron Microscopy

The first challenge of electron microscopy of liquid systems is making the liquid, quite often with a high vapor pressure system, compatible with high vacuum in the electron microscope (EM), as all EMs must operate under high vacuum. Older methods that were developed originally for biological research involve adding a “stain” to the system, often a salt or an acid of a heavy metal, and then drying the system to rid it of any volatiles. The stain improves image contrast and sometimes stabilizes the specimen. While this type of specimen preparation may work for some stable aggregates in liquid systems, addition of a salt or an acid to the system changes its ionic strength or pH, and drying affects its concentration, all of which most likely change the original nano- and microstructure of the system [45,46]. The alternative is to cool the system to a temperature at which its viscosity is very high, e.g., to the glassy or solid state, at which its vapor pressure is much lower than the pressure in the EM column and all supramolecular motion is arrested [47,48]. The preferred state is the glassy state, namely, when the liquid is cooled to a glass-like supercooled state without a phase transformation into a crystalline solid. This process of forming a glassy state, i.e., vitrification, prevents structural artifacts following solute segregation during freezing, which forms artifactual aggregates, and electron optical artifacts, the result of interaction of the electron beam with the crystalline specimen and defects on it [49,50].

Fast cooling of the specimen and performing electron microscopy while the specimen is maintained at a very low (cryogenic) temperature are the basis of cryogenic-temperature electron microscopy (cryo-EM), which can be performed as transmission electron microscopy (cryo-TEM) or as scanning electron microscopy (cryo-SEM). Vitrification requires very high cooling rates. To vitrify water, one needs to cool it at a rate of at least 10^5^ K/s [51]. To vitrify most organic solvents, a cooling rate of about 7000 K/s has been found to be sufficient [52].

Another major issue in cryo-EM is preparing the specimen under controlled conditions so that its temperature and concentration do not change during the process and the specimen imaged by cryo-EM faithfully represents the bulk system from which it is drawn [53]. Furthermore, mechanical manipulation of the liquid to give the specimen its final geometry may involve shear and elongation, which may lead to flow-induced artifacts; in some cases, those can be overcome by relaxation of the liquid in its final geometry before it is vitrified [54].

Once the cryo-specimen has been prepared, it is transferred under controlled conditions into an EM to protect it from heating and frost deposition from exposure to the ambient air. In the microscope, it is kept at the cryogenic temperature in a special cryo-holder designed for the particular instrument.

Imaging cryo-EM specimens is, quite often, more complicated than imaging room-temperature specimens. Most cryo-EM specimens are more sensitive to damage by the electron beam during imaging due to the juxtaposition of water and organic materials in such specimens [55]. That requires special care to minimize electron exposure of the specimen before a micrograph is recorded. Another issue, typical of many cryo-EM specimens, is the low image contrast between different domains of the specimens. That is especially true in systems made mostly of light elements, namely, hydrogen, carbon, and oxygen, and, in some cases, of somewhat heavier ones, such as phosphorous, sulfur, and sodium [56].

Earlier publications by our research group and by others described the technical details connected with the issues mentioned above. Here, we give only the basic technical points involved in cryo-TEM and cryo-SEM, referring the reader to the relevant literature and references therein [37,49,50,53,57,58,59].

### 2.1. Cryogenic-Temperature Transmission Electron Microscopy (Cryo-TEM)

TEM specimens must be thin, typically thinner than 300 nm, to limit inelastic electron scattering, which adds only noise, no information, to the image. The currently standard way to prepare vitrified cryo-TEM specimens of liquids was first suggested by Dubochet et al. [47]. It is based on applying a small drop, ca. 3 μL, to a perforated film supported on a standard TEM grid. The film can be either a perforated carbon-coated polymer film with holes of random size (around 1 μm) and shape or a thin silicon film in which uniform holes are drilled. Both types of perforated film-covered grids are available commercially. The drop is then blotted into a thin liquid film spanning the holes by bringing it into contact with a piece of filter paper [53]. This entire process should be performed in a chamber, e.g., that of the Controlled Environment Vitrification System (CEVS) [53], in which the temperature and saturation of the gas phase are controlled. The use of a CEVS prevents nanostructural changes in the liquid due to changes of temperature and changes in its concentration by evaporation or condensation [53]. In some cases, specimen preparation should be carried out in an inert atmosphere of dry air or nitrogen [60]. Commercial systems that follow the original concept of the CEVS are now commercially available in automated versions [61,62]. However, unlike the original CEVS, they cannot be used to prepare cryo-SEM specimens, systems originally at temperatures below room temperature, or non-aqueous systems.

In the course of drop thinning during specimen preparation, the liquid may be subjected to high rates of shear and elongation that could induce nanostructural changes in the system [54]. Those can be reversed by letting the liquid relax. In most cases, relaxation for 30–90 s is sufficient. That can be done safely in the controlled atmosphere of the preparation chamber.

After the thin liquid film is prepared and, if needed, relaxed, it is plunged mechanically into the proper cryogen at a speed on the order of 2 m/s. Aqueous films are typically plunged into freezing ethane, at −183 °C. Its relatively high boiling temperature of −90 °C prevents development of a gas film, which reduces the cooling rate, around the cooled specimen. The high surface area-to-volume ratio of the specimen, high-speed plunge, and the nature of the cryogen lead to the desired cooling rate of 10^5^ K/s needed for water vitrification [51]. Because liquid ethane is a good solvent for most organic liquids, it cannot be used as a cryogen for their vitrification. Fortunately, liquid nitrogen, which is inert, although a much less effective coolant, gives a sufficient cooling rate to vitrify most organic solvents, with the notable exception of linear hydrocarbons. More information on cryo-specimen preparation of nonaqueous systems can be found elsewhere [59].

Vitrified specimens may be stored under liquid nitrogen. For imaging, the specimen is loaded into a TEM cooling holder (“cryo-stage”) mounted in a cooled “transfer station”, and the holder is inserted into the TEM. The holder tip is kept at about −180 °C to avoid sublimation and to preserve the supercooled state of the vitreous ice. It is important to maintain clean high vacuum in the TEM provided by an oil-free vacuum system and a “cryo-box” around the specimen, namely, cryogenically cooled surfaces surrounding the specimen, minimizing condensation of volatiles on the specimen.

As stated above, imaging of soft matter cryo-specimens is complicated by inherent low image contrast and high sensitivity to electron beam radiation damage. The TEM accessory “Volta phase plate” acts in a similar manner to the Zernike glass phase plate used in light microscopy and similarly converts invisible phase differences into amplitude differences that are discernable by the camera and the human eye [63]. In EM of soft matter, we try to use the minimum electron dose possible, usually less than 10 e^–^/Å^2^, four to five orders of magnitude lower than that used in EM of “hard matter.” That is facilitated by the TEM software, which allows us to record images of the area not previously exposed to the beam. Modern direct imaging cameras, in which no intermediate step of scintillation is involved, are also important in allowing to record high-resolution images at very low electron exposure.

### 2.2. Cryogenic-Temperature Scanning Electron Microcopy (Cryo-SEM)

While TEM are true optical microscopes, SEM are scanning probe microscopes, forming a magnified picture of the specimen, pixel-by-pixel, each one with the brightness corresponding to the strength of the signal collected from a point in the specimen as the scanning electron beam interacts with it. The most useful signals for SEM imaging are secondary and backscattered electrons. The signals are collected by dedicated detectors mounted either outside or inside the SEM column. Another important signal consists of the X-rays emitted from the specimen following ionization of inner shell electrons of the specimen atoms by the electron beam. The energy of these X-rays is characteristic of the element from which they are emitted. Proper selection of the detector type, like all other parameters of operating a SEM, especially the electron acceleration voltage and the position of the specimen, is of paramount importance to obtaining the required structural and elemental composition information.

Cryo-SEM complements cryo-TEM, as it lets one examine dispersions of larger than μm-sized objects, in which nm-scale details are to be resolved. Furthermore, it can be used to image very viscous liquids that cannot be made into thin cryo-TEM specimens. While specimen preparation is different because cryo-SEM specimens are typically much thicker than those of cryo-TEM, the need to prepare them under controlled temperature and saturation conditions is the same in both methodologies. Thus, our research group used the original concept of the CEVS and modified it for cryo-SEM specimen preparation [64]. That paper also describes the preparation of these specimens, on a single metal carrier or sandwiched between two such carriers. So far, no commercial equivalent of this cryo-SEM CEVS is available. The cooling rates attained by plunging such specimens even into the best cryogen are insufficient to vitrify the liquid because of their geometry. That can be overcome by high-pressure freezing (HPF) [65,66], namely, by cooling the specimen while it is subjected to high pressure, about 200 MPa, of liquid nitrogen, which slows nucleation and growth rate of ice crystals. A comparison of cryo-SEM specimen preparation by plunging and by HPF in the study of EVs released from monocytes was performed by Koifman et al. [8]. HPF is performed using commercially available equipment, such as the Leica EM ICE system [67]. After thermal fixation, the specimen is fractured, either by a cooled knife to remove the upper part of the frozen drop or by split-opening the frozen sandwich held in a dedicated “specimen table.” That is usually performed as the specimen is mounted on a cold stage to keep it at cryogenic temperature and under vacuum, as, for example, in the Leica EM ACE900 system [68]. That system allows one to remove some of the vitrified liquid by sublimation to enhance contrast. It can also be used to coat the specimen with metal or carbon, either to prevent specimen charging of the nonconductive specimen or to form a freeze-fracture replica [69]. We prefer not to use the latter methodology as cryo-SEM has made so much progress in the last decade.

Sublimation of volatiles to enhance topographic specimen contrast should be performed with care as raising the temperature to reach a sufficiently high vapor pressure, e.g., above −120 °C for water, may lead to a temperature where ice crystals grow at a rate that can lead to nanostructural changes. Regarding coating a nonconductive specimen, if the proper acceleration voltage is used in the SEM, nonconductive specimens maintain their electrical neutrality [56], making coating unnecessary, which gives better resolution and saves labor in preparing the cryo-specimen.

The transfer of the cryo-specimen between the above steps of preparation is performed with a cooled, evacuated shuttle. At the end of the process, the specimen in its specimen table is transferred onto the cryo-stage of the SEM through an airlock. The specimen is kept at a temperature of about −150 °C. Clean vacuum in the SEM is critical for keeping cryo-specimen surface contamination-free during the microscopy session.

For high-resolution SEM, low electron acceleration voltage, on the order of 1 kV, should be used to limit beam spread in the specimen. As it turns out, acceleration voltage in this range is also needed to keep neutral even uncoated, nonconductive specimens (see above). It should be noted that only field emission electron guns (FEGs) can provide sufficient brightness at such low acceleration voltages. Also, at such low acceleration voltages, the probability of ionization events is higher than at higher acceleration voltages, which makes it necessary to apply low-dose protocols to minimize the number of electrons used for recording the micrograph. Unlike in TEM, we are not familiar with automatic, computer-controlled protocols to perform low-dose SEM.

To obtain most information from a cryo-SEM specimen, the user should optimize the microscope parameters and use the proper combination of detectors. We demonstrate these points below. We give here several examples of cryo-EM micrographs of typical pharmaceutical applications. We use these to make several key points regarding the type of information one can obtain from cryo-EM as applied to these systems. We also highlight some of the important practical issues regarding cryo-TEM and cryo-SEM.

## 3. Examples of Cryo-EM Applications

**Figure 1:**
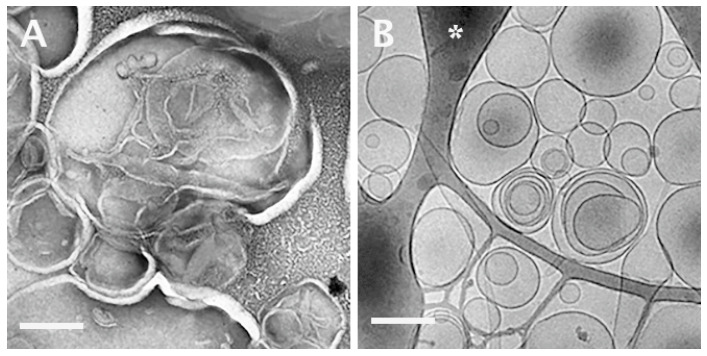
TEM images of the exogenous lung surfactant, Curosurf^®^: (**A**) a room-temperature specimen prepared by staining with a uranyl acetate solution and drying; (**B**) a cryo-TEM image of the same system. The dark domains in (**B**) are parts of the perforated carbon film (asterisk). Scale bars correspond to 200 nm. Adapted from [46].

Figure 1 shows a comparison between two specimens of the same system, namely, that of the exogenous lung surfactant, Curosurf^®^. One specimen was prepared by staining and drying (Figure 1A), and the other by cryo-TEM (Figure 1B) [46]. The former was made by applying a 5-μL drop of the sample solution to a continuous carbon film supported in a 200 mesh TEM copper grid, adding a drop of a 2 wt.% solution of uranyl acetate, gently blotting most of the liquid away, and letting it dry overnight. The cryo-specimen was prepared as described elsewhere [53] and imaged using a Philips CM120 TEM (Eindhoven, The Netherlands) at 120 kV. The specimen was equilibrated at −180 °C in an Oxford CT-3500 cooling holder (Abingdon, UK); it was imaged at low electron exposure of less than 20 e^–^/Å^2^ to minimize electron beam radiation damage. The image was recorded using a Gatan 791 MultiScan CCD camera at an underfocus of 7 μm to enhance phase contrast. The much better preservation of the Curosurf^®^ liposomes is obvious. Their walls are intact. The matrix in which the liposomes are embedded in the specimen and their interiors are made of vitrified water (“amorphous ice”).

**Figure 2:**
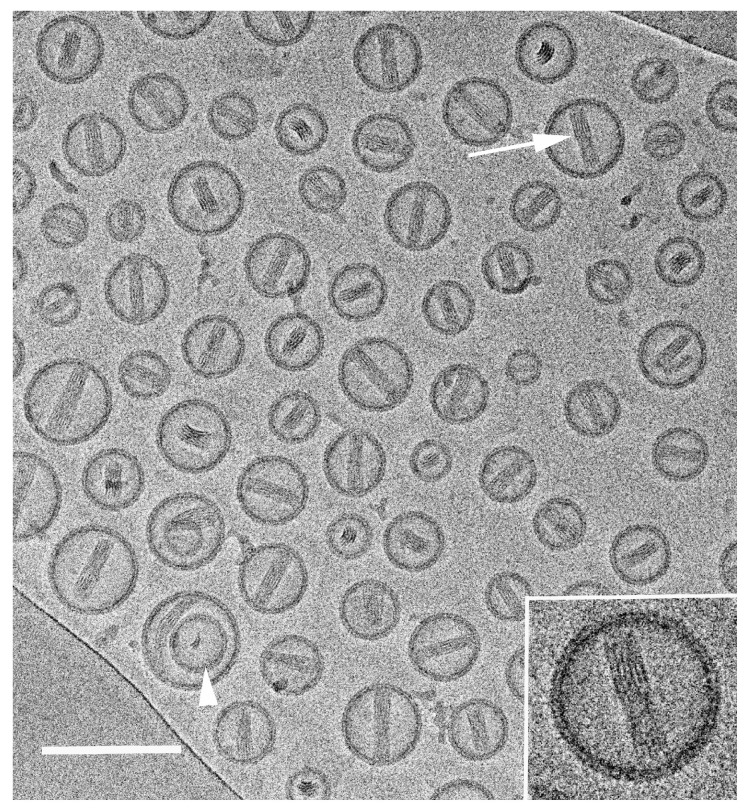
A cryo-TEM image of Doxil^®^. The arrow points to a doxorubicin crystal encapsulated inside a single-membrane liposome. The same particle is shown at a higher magnification in the inset. The arrowhead points to an empty vesicle embedded in another one. The scale bar corresponds to 100 nm.

Figure 2 shows a cryo-TEM image of Doxil^®^. This image was taken with a Talos 200C FEG-equipped high-resolution TEM (Thermo Fischer Scientific, formerly FEI; instrument assembled in the Czech Republic). Image contrast was enhanced with a Volta phase plate, and the image was recorded with a Falcon III direct imaging camera manufactured by the same company. The exposure was less than 20 e^–^/Å^2^. The arrow points to a doxorubicin crystal (the cytotoxic drug) encapsulated inside a single-membrane liposome. The same particle is shown at a higher magnification in the inset. The crystalline structure of the encapsulated crystal is resolved clearly. The part of the crystal that appears featureless suggests that the crystal is twisted. The bilayer structure of the membrane is well-resolved. Another liposome, indicated with an arrowhead, encapsulated a smaller vesicle and a doxorubicin crystal.

Images like this one provide important information about the size distribution of vesicles and the nature of their membrane. In this case, the membranes were intact, suggesting excellent encapsulation. The membranes were smooth, indicating that the lipids were at a temperature above the melting point of their hydrocarbon chains. When membranes have cusps, this indicates chains that are in the frozen state [70]. The vesicles are “stealth” vesicles, invisible to the body’s immune systems, shielded by polyethylene glycol chains grafted to some of the lipid molecules. Those water-miscible polymer chains are not visible by cryo-TEM due to lack of image contrast.

Cryo-SEM can provide analysis of a fractured frozen specimen by a combination of four detectors: a standard Everhart–Thornley (ET) secondary electron imaging (SEI; “SE2” in the Zeiss lingo) detector, an in-the-column secondary electron imaging (SEI, “InLens”) detector, an in-the-column backscattered electron imaging (BEI; “EsB”) detector, and an X-ray energy detector for energy-dispersive X-ray spectroscopy (EDS). The ET detector provides mostly topographical information due to the combination of secondary electron signals with some backscattered electrons that give a shadowing effect, which leads to an apparent 3D appearance of the micrograph. The “InLens” detector is used for high-resolution SEI; it lacks the contribution of backscattered electrons. In the micrographs generated by the EsB detector, there is almost no topographical information. The contrast is almost entirely based on the elemental composition of the specimen. At low electron acceleration voltage, on the order of 1 kV, the BEI contrast between light elements of very close atomic numbers is quite appreciable, so that even carbon (atomic number Z = 6) features good contrast relative to oxygen (Z = 8) [56]. That gives, for example, good contrast between water and oil, which is of paramount importance for biological and pharmacological systems.

The EDS detector system sorts the X-ray photons emitted from the specimen according to their energy. That information may be displayed as an energy spectrum. In addition, the signal specific to a given element may be used to map the relative concentration of that element in the specimen. The signals from several elements can be displayed superposed on a micrograph to display the relative concentration of several elements in a given area of the specimen.

**Figure 3:**
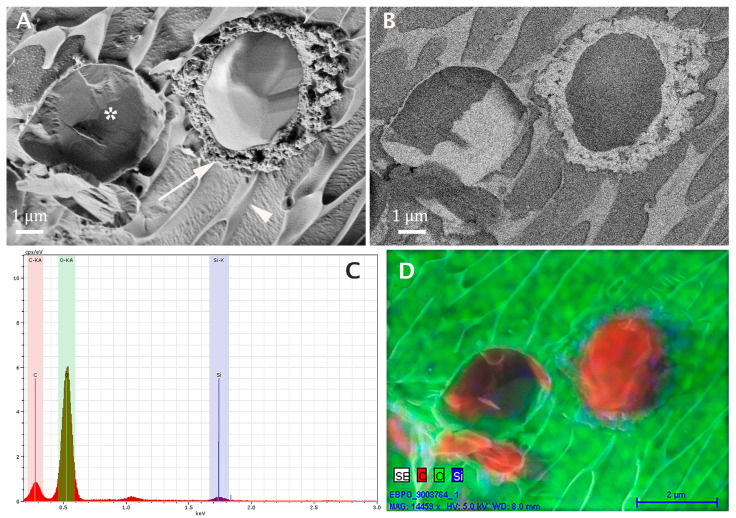
Cryo-SEM of BPO silica microcapsules. (**A**) A secondary electron imaging micrograph of a fractured frozen specimen. The capsule on the right was fractured through, showing very clearly the silica shell (arrow) around the oil phase core. The particle on the left (asterisk) was not fractured completely. The background is the frozen aqueous phase. The arrowhead points to a grain boundary. (**B**) Same area as in A recorded with the in-the-column backscattered electron imaging detector. The silica appears as the brightest domain, the water darker, and the oil darkest. (**C**) The EDS spectrum for the area imaged in (**A**,**B**). The C, O, and Si peaks are marked with different colors. The small peak at about 1.15 keV is of Na. (**D**) An elemental map using the same color labeling as in (**C**) shows the oxygen-rich water (green), the carbon-rich cores (reddish brown), and the Si-rich shells of the microcapsules (blue).

Figure 3 shows an example of a full cryo-SEM analysis of microcapsules of benzoyl peroxide (BPO) encapsulated in silica by the sol–gel process [2]. Figure 3A is a secondary electron imaging (SEI) micrograph. The microcapsule on the right was fractured through, exposing the core of the drug-containing oil and showing very clearly the silica shell (arrow) surrounding the core. As for the particle on the left, the fracture plane went partially above it, showing part of its outer shell (asterisk). The background is the frozen aqueous phase. Freezing leads to segregation of solutes from the forming ice crystals and their accumulation at grain boundaries (arrowhead). The same area shown in Figure 3A is recorded with the in-the-column backscattered electron imaging detector in Figure 3B. Here, the silica gives the brightest domains, the water domains are seen darker, and the oil core appears the darkest.

The EDS spectrum from the area imaged in A and B is given in Figure 3C. The C, O, and Si peaks are marked by different colors. The small peak at about 1.15 keV is of Na. This information is used for the elemental map given in Figure 3D. The colors of the peaks of Figure 3C are used here to mark the oxygen-rich water (green), the carbon-rich cores of the microcapsules (reddish brown), and the Si-rich shells of the microcapsules (blue).

**Figure 4:**
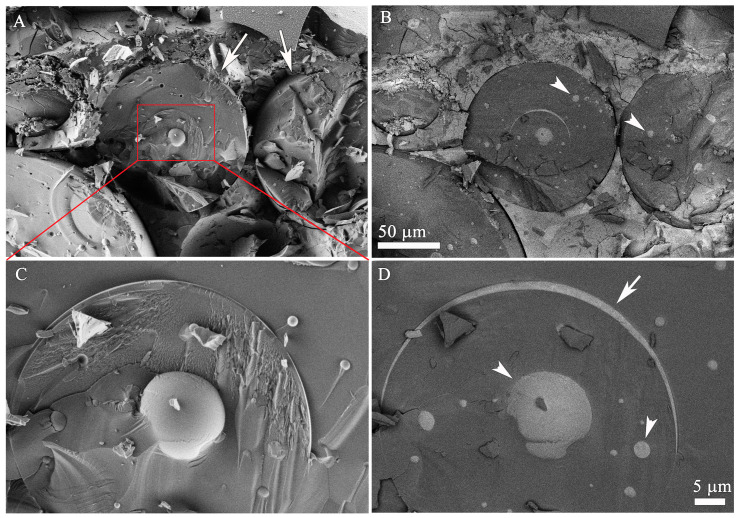
Comparison of the use of ET SEI (**A**,**C**) vs. the EsB (**B**,**D**) detector at 1.4 kV in cryo-SEM micrographs of a multiple O/W/O/W emulsion. (**C**,**D**) show high magnification of the red square area in (**A**). The arrows in (**A**) point at fractured oil droplets. The white arrowheads in (**B**) show water droplets inside the fractured oil droplets. The white arrow in (**D**) points at a thin water layer separating the outer and the inner oil droplets. The white arrowheads in (**D**) show water droplets inside the inner oil droplet. The micrographs were acquired at a temperature of −145 °C. The specimen work distance, WD, was 2.5 mm. Reproduced with permission from Ultramicroscopy [56], 2020.

Figure 4 demonstrates the excellent contrast one can obtain in cryo-SEM between the oil and water domains by using the appropriate acceleration voltage and detector. Here, we examined a high internal phase emulsion (HIPE) prepared by emulsifying 85% mineral oil (Shell Catenex Oil S 579) into a 0.6% (w/w) hexaethylene glycol monododecyl ether (C_12_E_6_) (Anatrace, Holland, OH, USA) solution with a SpeedMixer DAC 150.1 (Haushild Engineering, Hamm, Germany) at 3000 rpm for 20 min.

We prepared cryo-SEM specimens of the emulsions using the drop plunging method as described in detail elsewhere [56]. The cryo-specimens were studied using the same Zeiss Ultra Plus HR-SEM as in the previous example. We compared the micrographs recorded with the ET and EsB detectors (see above). The electron acceleration voltage was optimized at 1.4 kV. Note that the micrograph contrast is very sensitive to the acceleration voltage, and in some cases very low contrast and reversal of contrast are observed as a function of the beam acceleration voltage [56]. At the optimized voltage, the uncoated cryo-specimens did not charge up, and the contrast between the water and oil domains in the backscattered electron imaging micrographs was found to be the highest for this system.

As shown in Figure 4, the contrast in the ET micrographs (Figure 4A,C) was mostly due to the topography of the fractured surface, so one cannot tell the oil from the water domains. When the same area of the fractured specimen was imaged with the EsB detector (Figure 4B,D), the oxygen-rich water domains appeared much lighter than those of the carbon-rich oil. That allows one to show clearly the true nature of this oil-in-water-in-oil-in-water (O/W/O/W) multiple emulsion. In some cases, only a very thin layer of water and surfactant (arrow in Figure 4D) separates an inner oil domain from the outer domain.

## 4. Concluding Remarks

Cryo-EM is essential for full characterization of any liquid complex system. That is also true for any pharmaceutical liquid system used either for therapeutic or diagnostic purpose. This group of methodologies allows us to identify all the coexisting types of aggregates in the liquid phase which are difficult to obtain using indirect non-imaging methodologies, such as scattering techniques, rheology measurements, and nuclear magnetic resonance (NMR) techniques. The current state of the cryo-TEM and cryo-SEM imaging technologies at the disposal of researchers and technologists is a toolbox from which one needs to choose the proper tool taking into account the nature of the system and the type of information that is required. Judicious selection of the tools includes the type of methodology and operating parameters of the instrument, such as the type of the detector or detectors and the electron acceleration voltage to be used. That requires good understanding of the physics of electron beam–specimen interactions, and in particular the way micrographs are formed and recorded, micrograph contrast, and damage to the specimen by the beam.
